# Zafirlukast Is a Dual Modulator of Human Soluble Epoxide Hydrolase and Peroxisome Proliferator-Activated Receptor γ

**DOI:** 10.3389/fphar.2019.00263

**Published:** 2019-03-20

**Authors:** Tamara Göbel, Olaf Diehl, Jan Heering, Daniel Merk, Carlo Angioni, Sandra K. Wittmann, Estel.la Buscato, Ramona Kottke, Lilia Weizel, Tim Schader, Thorsten J. Maier, Gerd Geisslinger, Manfred Schubert-Zsilavecz, Dieter Steinhilber, Ewgenij Proschak, Astrid S. Kahnt

**Affiliations:** ^1^Institute of Pharmaceutical Chemistry/ZAFES, Goethe University Frankfurt, Frankfurt am Main, Germany; ^2^Branch for Translational Medicine and Pharmacology, Fraunhofer Institute for Molecular Biology and Applied Ecology IME, Frankfurt am Main, Germany; ^3^Faculty of Medicine, Institute of Clinical Pharmacology, Pharmazentrum Frankfurt, ZAFES, Frankfurt am Main, Germany; ^4^Department of Anesthesiology, Intensive Care Medicine and Pain Therapy, University Hospital Frankfurt, Goethe University Frankfurt, Frankfurt am Main, Germany

**Keywords:** PPARγ, soluble epoxide hydrolase, zafirlukast, montelukast, pranlukast, metabolic syndrome, polypharmacology

## Abstract

Cysteinyl leukotriene receptor 1 antagonists (CysLT1RA) are frequently used as add-on medication for the treatment of asthma. Recently, these compounds have shown protective effects in cardiovascular diseases. This prompted us to investigate their influence on soluble epoxide hydrolase (sEH) and peroxisome proliferator activated receptor (PPAR) activities, two targets known to play an important role in CVD and the metabolic syndrome. Montelukast, pranlukast and zafirlukast inhibited human sEH with IC_50_ values of 1.9, 14.1, and 0.8 μM, respectively. In contrast, only montelukast and zafirlukast activated PPARγ in the reporter gene assay with EC_50_ values of 1.17 μM (21.9% max. activation) and 2.49 μM (148% max. activation), respectively. PPARα and δ were not affected by any of the compounds. The activation of PPARγ was further investigated in 3T3-L1 adipocytes. Analysis of lipid accumulation, mRNA and protein expression of target genes as well as PPARγ phosphorylation revealed that montelukast was not able to induce adipocyte differentiation. In contrast, zafirlukast triggered moderate lipid accumulation compared to rosiglitazone and upregulated PPARγ target genes. In addition, we found that montelukast and zafirlukast display antagonistic activities concerning recruitment of the PPARγ cofactor CBP upon ligand binding suggesting that both compounds act as PPARγ modulators. In addition, zafirlukast impaired the TNFα triggered phosphorylation of PPARγ2 on serine 273. Thus, zafirlukast is a novel dual sEH/PPARγ modulator representing an excellent starting point for the further development of this compound class.

## Introduction

In the last decades, the main intention of rational drug discovery was the design of selective ligands, following the paradigm “one drug – one target – one disease.” However, in most cases drugs interact with a multitude of targets. These interactions referred to as the polypharmacological profile of a drug can contribute either to adverse reactions or pleiotropic effects thus synergistically enhancing the efficacy of a compound (Peters, 2013). The latter is of special interest for the treatment of complex multifactorial pathophysiological conditions such as inflammation. Arachidonic acid metabolites such as leukotrienes, prostaglandins and lipoxins are important players in initiation and resolution of inflammation and the majority of anti-inflammatory drugs interfering with eicosanoid signaling are fatty acid mimetics (Proschak et al., 2017). The polypharmacology of these non-steroidal anti-inflammatory drugs has been extensively reviewed in the past and it was concluded that addressing multiple inflammatory pathways is in general beneficial for overall efficacy and safety of these anti-inflammatory compounds (Hwang et al., 2013; Meirer et al., 2014; Reker et al., 2014).

**FIGURE 1:**
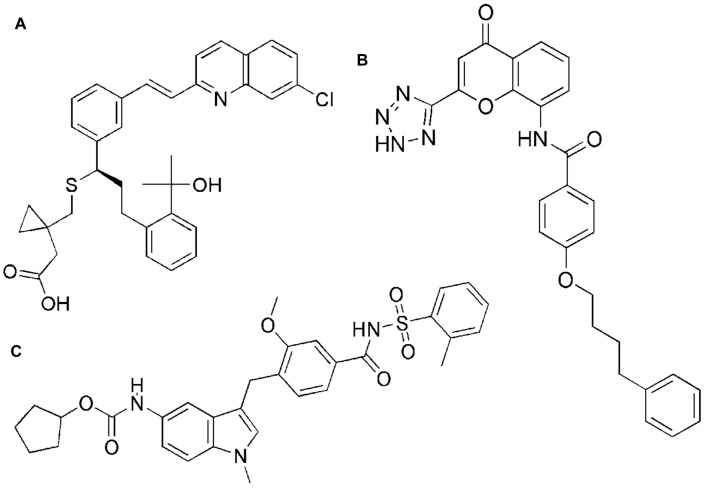
Chemical structures of montelukast **(A)**, pranlukast **(B)**, and zafirlukast **(C)**.

The cysteinyl leukotriene receptor 1 antagonists (CysLT1RA) montelukast, zafirlukast and pranlukast (Figure 1) were initially developed for the treatment of asthma. Displaying low nanomolar inhibitory activity to their target receptor these well-tolerated compounds were designed to inhibit the vasodilatory and bronchoconstrictory activities of cysteinyl leukotrienes. In addition to their anti-inflammatory properties in asthma they were found to be effective in different animal models among them studies of chronic obstructive pulmonary disease, atherosclerosis and the metabolic syndrome (MetS). In line with this, asthmatic patients taking montelukast display lower levels of cardiovascular disease (CVD)-associated inflammatory biomarkers and blood lipids. Moreover, montelukast appears to reduce the risk for recurrent stroke and myocardial infarction (Ibrahim et al., 2014; Theron et al., 2014; Hoxha et al., 2017). Indeed, we and others could recently show that the marketed CysLT1RA display an interesting polypharmacological profile by inhibition of additional pro-inflammatory targets such as microsomal prostaglandin E_2_ synthase-1 (mPGES-1), 5-lipoxygenase (5-LO), cAMP phosphodiesterases and NFκB (Ramires et al., 2004; Anderson et al., 2009; Woszczek et al., 2010; Fogli et al., 2013; Kahnt et al., 2013; Theron et al., 2014). Nevertheless, these already published off-target effects do not sufficiently explain their efficacy seen in models of atherosclerosis and the MetS.

The MetS is a multifactorial disease cluster consisting of central obesity, dyslipidemia, type 2 diabetes mellitus (T2D) and the increased risk for CVD that is considered a major public-health challenge (Eckel et al., 2010). Affected patients are at high risk to develop T2D and cardiovascular complications (Alberti et al., 2009; Kaur, 2014). Currently, the first-line treatment of the MetS focuses on the treatment of the accumulating risk factors. This results in a rising number of different medications increasing the pharmacologic complexity due to poorly predictable drug-to-drug interactions and raising therapy costs (Grundy, 2006). Among the frequently prescribed medications thiazolidinediones (TZD), peroxisome proliferator activated receptor γ (PPARγ) activating drugs, are used to reduce insulin resistance. Unfortunately, their clinical use is limited due to excessive weight gain, fluid retention and increased osteoporosis risk. Another drawback is the poor effect of TZDs on the occurrence of macrovascular events, although the equilibration of blood glucose levels reduces microvascular complications (Rohatgi and McGuire, 2008). Interestingly, PPARγ agonism combined with soluble epoxide hydrolase (sEH) antagonism shows the potential for a beneficial outcome in terms of macrovascular events (Drew et al., 2008; Imig et al., 2012; Xu et al., 2013; Hye Khan et al., 2018). Being part of the arachidonic acid cascade, endothelial sEH promotes the hydrolysis of vasorelaxing, cytochrome P450 derived epoxyeicosatrienoic acids (EETs) to the less bioactive corresponding dihydroxyeicosatrienoic acids (DHETs) (Imig and Hammock, 2009). Thus, the increase in circulating EET levels by inhibition of sEH is vasoprotective and EET linked effects on MetS-associated disorders including CVD, dyslipidemia, diabetic neuro- and nephropathy were already shown in various studies (Fleming, 2014).

Due to the protective effects of the CysLT1RA montelukast, zafirlukast and pranlukast seen in models of CVD we aimed at investigating their influence on human sEH and PPAR activities *in vitro*.

## Materials and Methods

### Cell Culture

3T3-L1 cells were obtained from the American Type Culture Collection (ATCC, Manassas, VA, United States). HEP-G2 and HEK-293T cells were bought from the Deutsche Sammlung für Mikroorganismen und Zellkulturen (DSMZ, Braunschweig, Germany). 3T3-L1 cells were cultured in DMEM containing 10% newborn calf serum, 1% sodium pyruvate (SP) and 1% penicillin/streptomycin (PS). HEP-G2 cells were cultured in DMEM supplemented with 10% fetal calf serum (FCS), 1% non-essential amino acids, 1% SP and 1% PS. HEK-293T cells were cultured in DMEM containing 10% FCS, 1% SP, and 1% PS. All cell lines were grown in a humidified atmosphere at 37°C, 5% CO_2_.

### Adipocyte Differentiation

3T3-L1 cells were differentiated into adipocytes for 14 days according to the protocol of Zebisch et al. (2012). For this, cells were seeded in 6-well plates (2.5 × 10^6^/well) and differentiation was induced after 48 h by addition of a differentiation cocktail containing 1 μg/mL insulin, 0.25 μM dexamethasone and 0.5 mM isobutylmethylxanthine (IBMX) in DMEM supplemented with 10% FCS and 1% PS. In addition, potential PPARγ activators (1, 5, and 10 μM zafirlukast or montelukast) or DMSO were added and rosiglitazone (2 μM) was used as PPARγ positive control. After 2 days, medium was replaced by DMEM supplemented with 10% FCS, 1% PS and 1 μg/mL insulin for 2 more days. Afterwards, cells were kept for lipid droplet accumulation in DMEM containing 10% FCS and 1% PS until day 15. Media were replaced every other day.

For investigations on PPARγ phosphorylation at Ser273, cells were differentiated for 10 days according to Choi et al. (2011). Cells were seeded in 6-well plates (2.5 × 10^6^/well) and differentiation was induced after 48 h by addition of 5 μg/mL insulin, 1 μM dexamethasone and 0.5 mM IBMX in DMEM supplemented with 10% FCS and 1% PS at 37°C, 5% CO_2_. After 2 days, medium was replaced by DMEM supplemented with 10% FCS, 1% PS and 5 μg/mL insulin. Medium was changed every other day until day 10. Then, cells were treated with 1 or 10 μM zafirlukast or 2 μM rosiglitazone for 45 min in DMEM supplemented with 10% FCS and 1% PS. After this, 50 ng/mL TNFα was added and cells were incubated for additional 30 min. Afterwards, cells were harvested, lysed and Western Blotting was performed as described below.

### Oil Red O Staining

Differentiated 3T3-L1 cells were washed with PBS and subsequently fixed for 60 min with formaldehyde (4% in PBS). Afterwards, cells were thoroughly rinsed with 60% isopropanol and incubated with Oil Red O solution (0.3% in 60% isopropanol) for 120 min. This was followed by the removal of the staining solution and thorough rinsing of the cell layer with ultrapure water. Finally, the plates were dried and pictures of the stained cell layers were taken.

For quantification of the accumulated dye, 3T3-L1 cells were seeded in 24-well plates (0.55 × 10^5^/well) instead of 6-wells and the differentiation was carried out as described in the section ‘Adipocyte differentiation’. After 14 days of differentiation, cells were fixed and stained as described above. Afterwards, cells were incubated with 150 μl of dye extraction solution (5% IGEPAL CA 630 in 100% isopropanol) for 30 min while gently shaking. 100 μl of dye extract were transferred to a 96-well plate and absorbance was measured (λ_abs_ = 510 nm) by an Infinite F200 plate reader (Tecan Group Ltd., Männedorf, Switzerland). Blank wells were subtracted from samples. Values were normalized to the wells receiving the differentiation cocktail without a PPARγ agonist (w/o).

### *In vitro* Cell Viability Assay (WST-1)

For measurement of cell proliferation, 3T3-L1 cells were seeded in 24-well plates (0.55 × 10^5^/well) instead of 6-wells and the differentiation was carried out as described in the section ‘Adipocyte differentiation’. After 2 days of incubation with the differentiation cocktail with or without the PPARγ agonists, WST-1 reagent (Roche Diagnostic GmbH, Mannheim, Germany) was added (1:10) to the supernatant of the differentiating cells. Then, the cells were further incubated for 2 h at 37°C, 5% CO_2_ to allow color development. After this, cell supernatant absorbance was measured (λ_abs_ = 450 nm) and corrected to a reference wavelength (λ_abs_ = 690 nm) with an Infinite F200 plate reader (Tecan Group Ltd., Männedorf, Switzerland). After this, background absorbance was subtracted from all measurements and values were normalized to the differentiated control receiving the differentiation cocktail without addition of a PPARγ agonist (w/o).

### Protein Isolation and Western Blotting

Total 3T3-L1 or HEP-G2 cell lysates were prepared in lysis buffer (20 mM Tris-HCl, pH 7.4, 150 mM NaCl, 2 mM EDTA, 1% Triton X-100, 0.5% NP-40) supplemented with protease and phosphatase inhibitors (PhosSTOP^TM^ + cOmplete^TM^ Mini, Roche Diagnostics GmbH, Mannheim, Germany). Protein concentrations were quantified using the Pierce^TM^ BCA Protein Assay Kit (Thermo Scientific, Waltham, MA, United States). Total protein (30 μg/lane) was separated by SDS-polyacrylamide gel electrophoresis and transferred to nitrocellulose membranes (GE Healthcare Life Sciences, Little Chalfont, United Kingdom). Membranes were blocked with Odyssey blocking reagent (LI-COR Biosciences, Bad Homburg, Germany) for 1 h at room temperature. Ensuing, membranes were incubated with antibodies against CD36 (EPR6573, Abcam, Cambridge, United Kingdom), PPARγ (E-8, Santa Cruz Biotechnology, Heidelberg, Germany), FABP-4 (C-15, Santa Cruz Biotechnology, Heidelberg, Germany) or PPARγ Ser273 (Bioss Antibodies Inc., Woburn, MA, United States) overnight at 4°C. Afterwards, membranes were washed and incubated with fluorescence conjugated secondary antibodies (IRDye, LI-COR Biosciences, Bad Homburg, Germany). Protein antibody complexes were visualized on the Odyssey Infrared Imaging System (LI-COR Biosciences, Bad Homburg, Germany). β-actin (I-19, goat, polyclonal, Santa Cruz Biotechnology, Heidelberg, Germany) was used as loading control. The density of the immune reactive bands was analyzed using the Image Studio 5.2 software (LI-COR Biosciences, Bad Homburg, Germany).

### mRNA Isolation and Quantitative RT-PCR

3T3-L1 cells were lysed using TRIzol^®^ reagent (Ambion Life Technologies, Carlsbad, CA, United States). Subsequently, mRNA was isolated following the manufacturers protocol. DNA contaminations were digested using DNAse (DNase I, RNase-free Kit; Thermo Scientific, Waltham, MA, United States) and mRNA concentrations were determined using a NanoDrop^TM^2000 spectrophotometer (Thermo Scientific, Waltham, MA, United States). Afterwards, reverse transcription was performed using the High Capacity RNA-to-cDNA Kit (Applied Biosystems, Foster City, CA, United States) following the manufacturers protocol. PCR was performed with SYBR green fluorescent dye (Applied Biosystems, Foster City, CA, United States) with a StepOnePlus Real-Time PCR System (Applied Biosystems, Foster City, CA, United States) using specific primers for murine adiponectin, FABP-4, GLUT-4, and LPL (Table 1). Relative mRNA expression was determined by the 2^-ΔΔCt^ method normalized to murine non-POU domain-containing octamer binding protein (Nono). All samples were measured in triplicates and experiments were repeated independently at least three times.

**Table 1:** Primer sequences and nucleotide accession numbers of the genes investigated.

Murine gene	Nucleotide accession number	Primer sequence
FABP4	NM_024406.2	F: AGAAGTGGGAGTGGGCTTTG
		R: ACTCTCTGACCGGATGGTGA
SLC2A4 (GLUT4)	NM_009204	F: TGAAGAACGGATAGGGAGCAG
		R: GAAGTGCAAAGGGTGAGTGAG
LPL	NM_008509	F: CCCAGCTTCGTCATCGAGAG
		R: GTCCAGTGTCAGCCAGACTT
ADIPOQ (Adiponectin)	NM_009605	F: TGACGACACCAAAAGGGCTC
		R: CACAAGTTCCCTTGGGTGGA
Nono	NM_001252518.1	F: TGCTCCTGTGCCACCTGGTACTC
	NM_023144.2	
		R: CCGGAGCTGGACGGTTGAATGC


### PPAR Reporter Gene Assay

HEK-293T cells were seeded in 96-well plates (2.5 × 10^4^ cells/well) and were allowed to adhere overnight. Before transfection, medium was changed to Opti-MEM without supplements. Transient plasmid transfection was carried out using Lipofectamine LTX reagent (Invitrogen, Carlsbad, CA, United States) according to the manufacturer’s protocol with pFR-Luc (Stratagene, La Jolla, CA, United States), pRL-SV40 (Promega, Fitchburg, MA, United States) and pFA-CMV-PPAR-LBD (Rau et al., 2006). 5 h after transfection, medium was changed to Opti-MEM supplemented with penicillin (100 U/mL) and streptomycin (100 μg/mL), now additionally containing 0.1% DMSO and the respective test compound or 0.1% DMSO alone as untreated control. Each concentration was tested in triplicates and each experiment was repeated independently at least four times. Following overnight (12–16 h) incubation with the test compounds, cells were assayed for luciferase activity using the Dual-Glo^TM^ Luciferase Assay System (Promega, Fitchburg, MA, United States) according to the manufacturer’s protocol. Luminescence was measured with an Infinite M200 luminometer (Tecan Group Ltd., Männedorf, Switzerland). Normalization of transfection efficiency and cell growth was done by division of firefly luciferase data by renilla luciferase data and multiplying the value by 1000 resulting in relative light units (RLU). Fold activation was obtained by dividing the mean RLU of a test compound at a respective concentration by the mean RLU of untreated control. Relative activation was obtained by dividing the fold activation of a test compound at a respective concentration by the fold activation of the respective PPARα, γ or δ full agonist GW-7647, pioglitazone or L165,041 at 1 μM. EC_50_ and standard deviation were calculated with the mean relative activation values of at least four independent experiments by SigmaPlot 10.0 (Systat Software GmbH, Erkrath, Germany) using a four parameter logistic regression.

### Recombinant sEH Activity

For determination of IC_50_ values of recombinant human sEH a 96-well fluorescence-based assay system was used as described before (Blöcher et al., 2016a). In brief, non-fluorescent PHOME (3 phenylcyano-(6-methoxy-2-naphthalenyl)methyl ester 2-oxiraneacetic acid, Cayman Chemicals, Ann Arbor, MI, United States) was used as substrate, which is hydrolyzed by sEH to the fluorescent 6-methoxynaphthaldehyde (Rau et al., 2006). The formation of the fluorescent product was measured (λ_em_ = 330 nm, λ_ex_ = 465 nm) by an Infinite F200 Pro plate reader (Tecan Group Ltd., Männedorf, Switzerland). For that purpose, 2 μg recombinant human sEH in 100 μl bis-Tris buffer (pH 7, 0.1 mg/mL BSA, 0.01% Triton-X 100) was applied per well. This protein solution was then incubated with different concentrations of the test compounds for 30 min at RT. After this, 10 μL of the substrate were added (final concentration 50 μM). The formation of 6-methoxynaphthaldehyde was monitored for 30 min. All concentrations were tested in triplicate wells.

### sEH Activity in HEP-G2 Cell Lysates

Quantification of cellular sEH metabolic activity was performed as described by Zha et al. (2014). For this, HEP-G2 cells were harvested, washed twice with PBS and sonicated in PBS for disruption of cell integrity. Then, 1 μg of total cell homogenate diluted in 100 μl of PBS containing 0.1 mg/ml BSA was incubated with the compounds or vehicle for 15 min at 37°C. After this, 25 ng (±)14(15)-EET-d_11_ (Cayman Chemical, Ann Arbor, MI, United States) were added per sample and the incubation was continued for additional 10 min at 37°C. A blank was performed using PBS (containing 0,1 mg/mL BSA). The reactions were stopped by adding 100 μL of ice cold methanol. After centrifugation (2000 rpm, 4°C, 5 min), supernatants were analyzed by LC-MS/MS and the amounts of (±)14(15)-EET-d_11_ and the corresponding (±)14(15)-DHET-d_11_ were determined.

### Determination of (±)14(15)-EET-d_11_/(±)14(15)-DHETd_11_ by LC/MS-MS

14(15)-EET-d_11_ and 14(15)-DHET-d_11_ content of the extracted samples were analyzed employing liquid chromatography tandem mass spectroscopy (LC-MS/MS). The LC/MS-MS system comprised an API 5500 QTrap (Sciex, Darmstadt, Germany), equipped with a Turbo-V-source operating in negative ESI mode, an Agilent 1200 binary HPLC pump and degasser (Agilent, Waldbronn, Germany) and an HTC Pal autosampler (Chromtech, Idstein, Germany) fitted with a 25 μL LEAP syringe (Axel Semrau GmbH, Sprockhövel, Germany). High purity nitrogen for the mass spectrometer was produced by a NGM 22-LC/MS nitrogen generator (cmc Instruments, Eschborn, Germany). All substances were obtained from Cayman Chemical, Ann Arbor, MI, United States. Stock solutions with 2,500 ng/mL of both analytes were prepared in methanol. Working standards were obtained by further dilution with a concentration range of 0.1–250 ng/mL for 14(15)-EET-d_11_ and 14(15)-DHET-d_11_. Sample extraction was performed with liquid–liquid-extraction. Therefore, 150 μL of matrix homogenates were gently mixed with 20 μL of internal standard [14(15)-EET and 14(15)-DHET all with a concentration of 100 ng/ml in methanol], and were extracted twice with 600 μL of ethyl acetate. Samples for standard curve and quality control were prepared similarly, instead of 150 μL of matrix homogenates, 150 μL PBS were added. Further 20 μL methanol, 20 μL working standard and 20 μL internal standard were added. The organic phase was removed at a temperature of 45°C under a gentle stream of nitrogen. The residues were reconstituted with 50 μL of methanol/water/(50:50, v/v), centrifuged for 2 min at 10,000 *g* and then transferred to glass vials (Macherey-Nagel, Düren, Germany) prior to injection into the LC-MS/MS system. For the chromatographic separation a Gemini NX C18 column and pre-column were used (150 mm × 2 mm i.d., 5 μm particle size and 110 Å pore size from Phenomenex, Aschaffenburg, Germany). A linear gradient was employed at a flow rate of 0.5 mL/min mobile phase with a total run time of 17.5 min. Mobile phase was A water/ammonia (100:0.05, v/v) and B acetonitrile/ammonia (100:0.05, v/v). The gradient started from 85% A to 10% within 12 min. This was held for 1 min at 10% A. Within 0.5 min the mobile phase shifted back to 85% A and was held for 3.5 min to equilibrate the column for the next sample. The injection volume of samples was 20 μL. Quantification was performed with Analyst Software V 1.5.1 (Sciex, Darmstadt, Germany) employing the internal standard method (isotope- dilution mass spectrometry). Ratios of analyte peak area and internal standard area (*y*-axis) were plotted against concentration (*x*-axis) and calibration curves were calculated by least square regression with 1/concentration^2^ weighting.

### PPARγ Coactivator Recruitment Assay

Recruitment of coactivator-derived peptides to the PPARγ-LBD was studied by homogeneous time-resolved fluorescence resonance energy transfer (HT-FRET). Terbium cryptate as streptavidin conjugate (Cisbio assays, France) was used as FRET donor. Peptides derived from the coactivator cyclic AMP response element-binding protein (CREB)-binding protein (CBP) [biotin-NLVPDAASKHKQLSELLRGGSGS] encompassing the coactivator consensus motif LxxLL and N-terminal biotin for stable coupling to streptavidin were purchased from Eurogentec GmbH (Cologne, Germany). Solutions containing 12 nM recombinant PPARγ-LBD fused to N-terminal GFP as FRET acceptor and 12 nM FRET donor complex with the CBP-derived peptide as well as 1% DMSO with test compound at varying concentrations or DMSO alone were prepared in HEPES buffer [25 mM HEPES pH 7.5 adjusted with KOH, 150 mM KF, 5% (w/v) glycerol, 0.1% (w/v) CHAPS, 5 mM DTT]. After 2 h incubation at RT, the fluorescence intensities (FI) at 520 nm (acceptor) and 620 nm (donor reference) after excitation at 340 nm were recorded on a Tecan Infinite F200 (Tecan Group Ltd., Männedorf, Switzerland). FI_520nm_ was divided by FI_620nm_ and multiplied with 10,000 giving a dimensionless HTRF signal. Recruitment of coactivator-derived peptides to the PPARγ-LBD was validated with increasing concentrations of rosiglitazone. Recruitment of CBP was referenced to recruitment in response to 1 μM rosiglitazone (∼EC_80_) and reported as relative coactivator recruitment.

### Molecular Docking Studies

Molecular modeling experiments were carried out using MOE (Molecular Operating Environment v. 2016.0802, Chemical Computing Group, Montreal, QC, Canada). Structure preparation of PPARγ LBD (3WMH) and sEH c-terminal domain (5ALZ) were subjected to the Quick Preparation routine, which includes automated structure curation, determination of protonation state and restrained energy minimization. Afterwards, molecular docking of zafirlukast was performed using default settings for induced fit docking. London dG scoring function was used for initial placement of 30 poses and afterwards refinement was performed using the MM/GBVI method (Zha et al., 2014). After visual inspection, the highest-scored pose was used to put up the binding mode hypothesis.

### Drugs, Chemical Reagents and Other Materials

DMEM, OptiMEM, penicillin-streptomycin, PBS and sodium pyruvate were obtained from Gibco by Life Technologies (Carlsbad, CA, United States), SYBR green MicroAmp Fast Optical 96-well Reaction Plate 0.1 ml and StepOnePlus thermocycler were obtained from Applied Biosystems (Foster City, CA, United States). FCS was purchased from Capricorn (Ebsdorfergrund, Germany). New born calf serum was purchased from Biochrom (Berlin, Germany). All primer pairs were purchased from Eurofins (Friedrichsdorf, Germany). Cell culture flasks (T75, Cell+, vented cap) and 6-well plates for sub-culturing 3T3-L1 cells were obtained from SARSTEDT (Nümbrecht, Germany). Rosiglitazone, pioglitazone, zafirlukast, montelukast, IBMX and (±)14(15)-EET-d_11_ were purchased from Cayman Chemical (Ann Arbor, MI, United States). Insulin, dexamethasone and Oil Red O were obtained from Sigma Aldrich (St. Louis, Missouri, MO, United States). Tris, Triton-X-100, NP-40, NaCl, EDTA and SDS were purchased from AppliChem (Darmstadt, Germany).

### Statistical Analysis

All data are presented as mean with SEM (standard error of the mean). GraphPad Prism version 5.00 (GraphPad Software, San Diego, CA, United States) was used for statistical analysis. Data were subjected to either Repeated Measure Analysis Of Variance (ANOVA) coupled with Dunnett’s post *t*-test for multiple comparisons or a two-sided paired students *t*-test. A sigmoidal concentration-response curve-fitting model with a variable slope was employed to calculate the IC_50_ values [non-linear regression; dose-response-inhibition; log(inhibitor) vs. response- variable slope (4 parameters)].

## Results

### Influence of the CysLT1RA on the Activation PPAR Reporter Constructs

To investigate the influence of the three marketed CysLT1RA montelukast, pranlukast, and zafirlukast on the activation of the PPARs α, γ, or δ, a hybrid reporter gene assays specific for each isoform was utilized. For this, a hybrid receptor construct containing the respective human PPAR ligand binding domain and hinge region fused to the DNA binding domain of the yeast nuclear receptor Gal4 and a Gal4-responsive firefly luciferase construct were co-transfected into HEK-293T cells together with a plasmid constitutively expressing *Renilla* luciferase for normalization of transfection efficiency and cell growth. Activation of the PPAR isoforms was compared to 1 μM of the well characterized agonists GW7647, L165,041, and pioglitazone for PPARα, δ, and γ, respectively. PPARα and PPARδ were not activated by the CysLT1RA tested at concentrations up to 10 μM. In contrast, PPARγ was strongly activated by zafirlukast with an EC_50_ value of 2.49 ± 0.45 μM and a maximal activation of 148 ± 15% compared to pioglitazone (1 μM). Montelukast showed PPARγ activation as well, with an EC_50_ of 1.17 ± 0.08 μM but displayed only low overall activation of 21.9 ± 0.3%. Pranlukast only weakly activated PPARγ by 19.7 ± 1.1% at 10 μM and was therefore excluded from further characterization. Table 2 summarizes the results for activation of the different PPAR isoforms by the CysLT1RAs.

**Table 2:** Influence of the CysLT1RA on the activation of PPAR reporter constructs.

	PPARα	PPARγ	PPARδ	CysLT_1_R
		
	EC_50_ [μM]			IC_50_ [nM]
Zafirlukast	**i.a.** @ 10 μM	**2.49** ± 0.45 μM (148 ± 15%)	**i.a.** @ 10 μM	5
Montelukast	**i.a.** @ 10 μM	**1.17** ± 0.08 μM (21.9 ± 0.3%)	**i.a.** @ 10 μM	5
Pranlukast	**i.a.** @ 10 μM	**@ 10 μM** (19.7 ± 1.1%)	**i.a.** @ 10 μM	4


*Employing reporter gene assays for the respective PPAR isoforms, EC_*50*_ values of the CysLT1RA were determined in HEK-293T cells. PPAR activation of the positive controls GW-7647 (PPARα), pioglitazone (PPARγ) and L165,041 (PPARδ) were set to 100% and receptor activation of the CysLT1RAs were calculated accordingly. Results are shown as ±SEM of three independent experiments. i.a., inactive*.

### Influence of Zafirlukast and Montelukast on 3T3-L1 Adipocyte Differentiation

**FIGURE 2:**
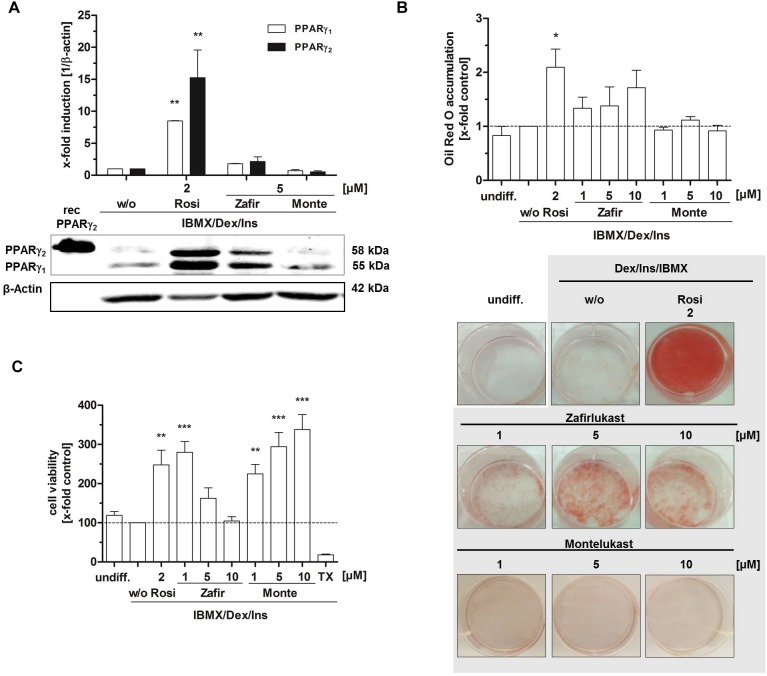
Influence of CysLT1RA on 3T3-L1 adipocyte differentiation. **(A)** Protein expression of PPARγ in 3T3-L1 adipocytes differentiated in the presence of 0.25 μM dexamethasone, 0.5 mM IBMX, 1 μg/ml insulin plus CysLT1RA or rosiglitazone (2 μM) or DMSO (w/o). One representative western blot out of three is shown. Photographs **(B)** and densitometric analysis of the lipid accumulation in 3T3-L1 adipocytes visualized by Oil Red O staining of the differentiated cells in the presence of montelukast and zafirlukast. Rosiglitazone and DMSO (w/o) were used as positive and negative control, respectively. One representative experiment out of three is shown. **(C)** WST-1 viability assay of 3T3-L1 cells treated with the compounds plus differentiation cocktail (0.25 μM dexamethasone, 0.5 mM IBMX, 1 μg/ml insulin) for 48 h. Ins, Insulin; IBMX, isobutylmethylxanthine; Monte, montelukast; Rosi, rosiglitazone; w/o, without PPARγ agonist; Zafir, zafirlukast. Significant changes versus the untreated control are indicated with an asterisk. ^∗^*P* ≤ 0.05, ^∗∗^*P* ≤ 0.01, ^∗∗∗^*P* ≤ 0.001.

As master regulator of adipocyte differentiation in men and mice, PPARγ plays a crucial role in the cell’s commitment to the adipocyte lineage by controlling important functions such as intracellular biosynthesis, transport and accumulation of lipids. Murine 3T3-L1 fibroblasts are a well characterized *in vitro* tool to study PPARγ induced adipocyte differentiation. These cells can be differentiated from fibroblasts into adipocytes by an activation cocktail (0.5 mM IBMX, 1 μg/mL insulin, 0.25 μM dexamethasone) together with the potential PPARγ agonist. Upon differentiation, different parameters such as lipid accumulation and PPARγ target gene expression can be assessed to investigate the PPARγ activating potential of the compounds of interest. Therefore, we treated confluent 3T3-L1 cell layers with the activation cocktail (IBMX, insulin, dexamethasone) plus different concentrations of zafirlukast or montelukast for 48 h to induce differentiation. Rosiglitazone (2 μM) was used as positive control. This was followed by insulin (1 μg/mL) treatment for another 48 h. After this, cells were kept in growth medium for a week to complete the differentiation process and allow intracellular lipid accumulation. Subsequently, the cells were stained with Oil Red O solution to investigate the extent of cytoplasmic lipid droplet accumulation as a measure for cell differentiation. As expected, the rosiglitazone (2 μM) control led to strong accumulation of lipid droplets in the cytoplasm of the cells indicated by intense Oil Red O staining. Interestingly, zafirlukast induced lipid accumulation in a dose-dependent manner, but to a lesser extend compared to rosiglitazone. Montelukast did not trigger any lipid accumulation in 3T3-L1 cells (Figure 2B). In line with the staining results, expression levels of both PPARγ isoforms γ_1_ and γ_2_ were not affected by montelukast whereas rosiglitazone and zafirlukast treatment upregulated the protein levels. Again, zafirlukast was less effective compared to rosiglitazone (Figure 2A).

In addition to lipid accumulation, mitochondrial activity of 3T3-L1 cells during differentiation was investigated by the turnover of the tetrazolium salt WST-1 into a formazan dye. This provides not only further information on the metabolic capacity of the cells after differentiation but can also indicate toxicity of compounds. For this, 3T3-L1 cells were treated with the differentiation cocktail in the presence or absence of PPARγ agonists for 48 h. After this, WST-1 turnover as a measure for mitochondrial activity was assessed (Figure 2C). As anticipated, undifferentiated 3T3-L1 cells as well as cells differentiated in the absence of a PPARγ agonist (w/o) displayed a third of the metabolic activity of cells treated with the PPARγ agonist rosiglitazone (2 μM). Montelukast potently elevated mitochondrial activity in a concentration dependent manner to over threefold of the w/o control at 10 μM. Also, cells treated with low concentrations of zafirlukast (1 μM) displayed potent metabolic activation (threefold) while higher concentrations (5 and 10 μM) showed attenuated metabolic activity which was comparable to the w/o control. Of note, microscopic analysis of the cell layers showed that this was not due to any cell loss, since the monolayer cultures were confluent in all treatments applied.

### Influence of Zafirlukast and Montelukast on PPARγ Target Gene Expression in Differentiated 3T3-L1 Adipocytes

**FIGURE 3:**
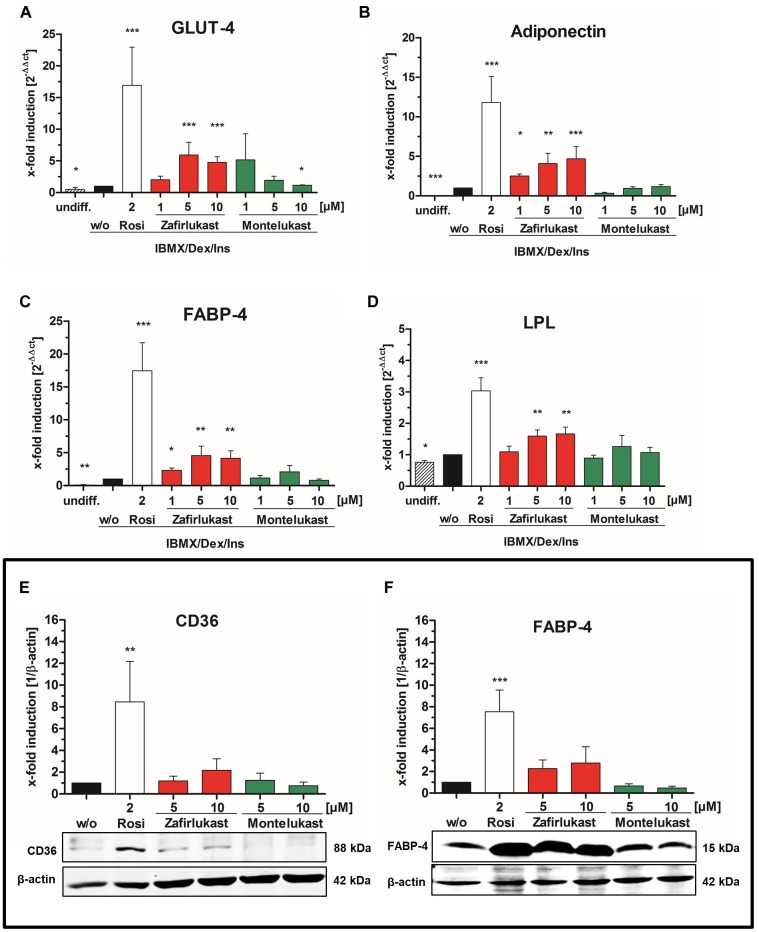
Influence of CysLT1RA on PPARγ target gene expression in 3T3-L1 adipocytes. Semi-quantitative mRNA expression of **(A)** GLUT-4, **(B)** adiponectin, **(C)** FABP-4, and **(D)** lipoprotein lipase in 3T3-L1 adipocytes differentiated in the presence of 0.25 μM dexamethasone, 0.5 mM IBMX, 1 μg/ml insulin plus the PPARγ agonist rosiglitazone (2 μM) or zafirlukast and montelukast. Results are shown as fold induction of the DMSO treated control (2^-ΔΔct^ method). Results are given as mean + SEM of three to six independent experiments. Western Blot experiments showing **(E)** CD36 and **(F)** FABP-4 protein expression in 3T3-L1 adipocytes differentiated in presence of 0.25 μM dexamethasone, 0.5 mM IBMX, 1 μg/ml insulin plus the PPARγ agonist rosiglitazone or zafirlukast or montelukast. DMSO (w/o) treated cells were used as negative control. Densitometry results are presented as mean + SEM. One representative western blot experiment out of three is shown. Significant changes versus the untreated control are indicated with an asterisk. ^∗^*P* ≤ 0.05, ^∗∗^*P* ≤ 0.01, ^∗∗∗^*P* ≤ 0.001.

To gain further insight in PPARγ activation after compound treatment, mature 3T3-L1 adipocytes were harvested after 14 days and mRNA was isolated, reverse transcribed and subjected to quantitative RT-PCR. mRNA expression of the PPARγ target genes lipoprotein lipase (LPL), the glucose transporter 4 (GLUT-4), the fatty acid binding protein 4 (FABP-4) and adiponectin was monitored by quantitative PCR (Figure 3A–D and Supplementary Figure S1). Activation of gene transcription was normalized to cells treated with the activation cocktail only. Again, montelukast did not influence the expression of the PPARγ target genes investigated while zafirlukast dose-dependently induced target gene expression although to a lesser extent compared to rosiglitazone. Of interest, zafirlukast elevated adiponectin levels more effectively compared to the other target genes.

In addition to mRNA expression, the protein expression levels of the two PPARγ target genes FABP-4 and CD36 in differentiated 3T3-L1 cells after 14 days were investigated. For this, total protein lysates were separated by gel electrophoresis followed by Western Blotting (Figure 3E,F and Supplementary Figures S2A,B). As anticipated, 2 μM rosiglitazone upregulated the expression of all target genes investigated with high potency. Again, zafirlukast induced target gene expression as well but to a weaker extent and montelukast displayed no effect at all.

### Influence of Zafirlukast on PPARγ Phosphorylation and Cofactor Recruitment

**FIGURE 4:**
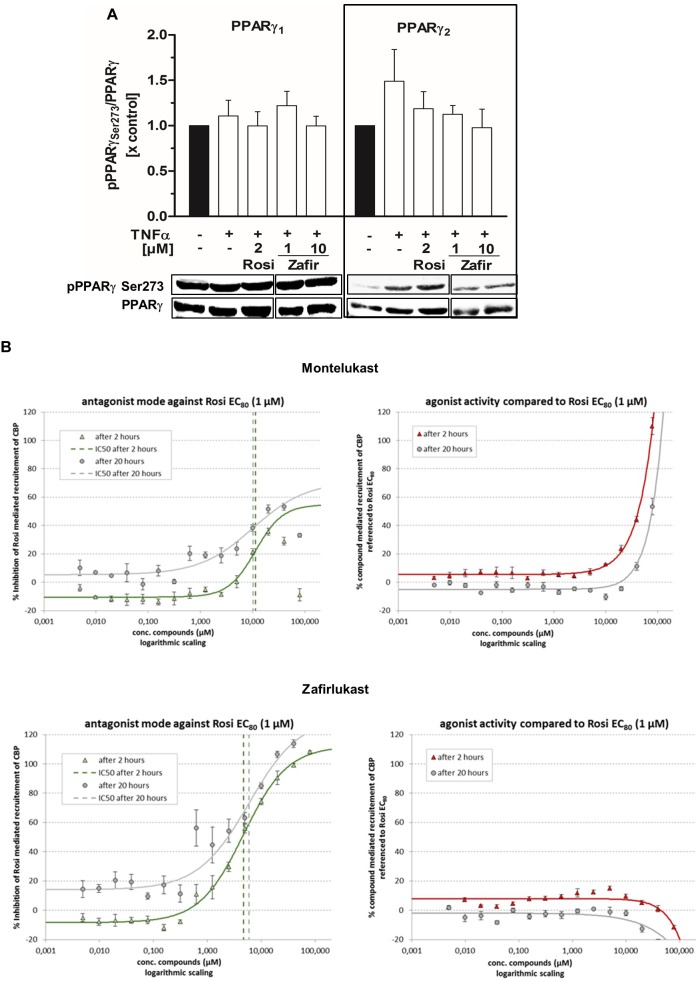
Influence of zafirlukast on PPARγ phosphorylation (Serine 273) and cofactor recruitment. **(A)** Differentiated 3T3-L1 adipocytes were treated with vehicle, the PPARγ agonist rosiglitazone or zafirlukast for 45 min followed by treatment with TNFα (50 ng/mL) for 30 min. Lysates of the treated cells were analyzed afterwards for PPARγ phosphorylation at Serine 273 by Western Blotting. Densitometry results are presented as mean + SEM. One representative Western Blot out of three is shown. **(B)** Recruitment of the recombinant cofactor CBP to the recombinant PPARγ-LBD by in presence and absence of rosiglitazone via time-resolved FRET analysis. Results are presented as mean ± SEM. Significant changes versus the untreated control are indicated with an asterisk. ^∗^*P* ≤ 0.05, ^∗∗^*P* ≤ 0.01, ^∗∗∗^*P* ≤ 0.001.

Next, we analyzed the ability of zafirlukast to interfere with the phosphorylation of PPARγ at serine 273 in 3T3-L1 cells. We induced this post-translational modification by short-term treatment of the differentiated cells with the cytokine TNFα, generating a pro-inflammatory milieu. In our hands, untreated cells already showed Serine 273 phosphorylation of PPARγ1 (uniprot ID: P37231-2) and this was not changed after addition of TNFα. In contrast, PPARγ2 (uniprot ID: P37231-1) was efficiently phosphorylated upon treatment with TNFα. The control compound rosiglitazone inhibited this phosphorylation, although this effect was not significant. Of note, zafirlukast inhibited this phosphorylation in our assay system as well (Figure 4A and Supplementary Figure S3). This indeed suggested an influence of zafirlukast on PPARγ cofactors. Thus, we evaluated the direct influence of montelukast and zafirlukast on the recruitment of coactivators to the receptor. For this, we used an assay system in which the recruitment of the fluorescence-tagged PPARγ coactivator CBP to the PPARγ-LBD was assessed by time-resolved FRET technique in presence and absence of rosiglitazone. Montelukast weakly antagonized the rosiglitazone induced CBP recruitment in concentrations above 3 μM and did not induce CBP recruitment itself in concentrations up to 20 μM. At higher concentrations montelukast seemed to trigger recruitment but this may be due to unspecific protein aggregation. Zafirlukast did not induce CBP recruitment itself. Instead, the compound was able to antagonize the rosiglitazone (1 μM) induced CBP recruitment with an IC_50_ of 4.7 μM after 2 h (Figure 4B).

### Influence of the CysLT1RA on sEH Activity

**FIGURE 5:**
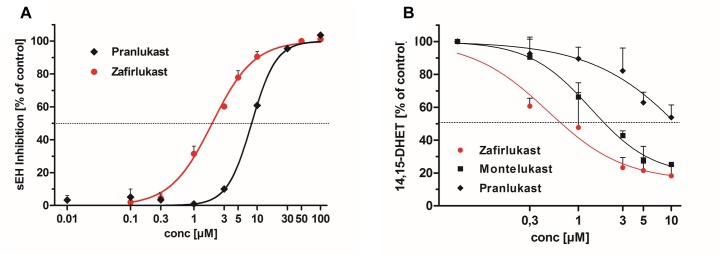
Influence of CysLT1RA on sEH activity. **(A)** Inhibition profiles of pranlukast and zafirlukast on recombinant human sEH. PHOME was used as fluorescence substrate. Results are given as mean + SEM of three independent experiments. **(B)** sEH inhibition profiles of pranlukast, montelukast and zafirlukast in HEP-G2 lysates. (±)14(15)-EET-d_11_ was used as sEH substrate and the resulting DHET levels where measured via LC-MS/MS. Results are given as mean + SEM out of three independent experiments.

In recent years, a number of animal studies could show that the combination of PPARγ activation with sEH inhibition is advantageous for the treatment of the MetS. Therefore, we were interested if the CysLT1RAs zafirlukast, pranlukast, and montelukast as eicosanoid mimetics might also interfere with sEH in addition to their PPARγ agonistic activities. For this, recombinant human sEH was incubated with zafirlukast or pranlukast in the presence of the non-fluorescent sEH substrate PHOME which is cleaved by the enzyme to form a fluorescent product. Montelukast was excluded from this assay due to its autofluorescence which interfered with product detection. Indeed, zafirlukast and pranlukast inhibited the recombinant sEH with an IC_50_ of 1.97 ± 0.08 μM and 8.86 ± 0.32 μM, respectively (Figure 5A and Table 3). To confirm these results and to determine the influence of montelukast on sEH activity, conversion of deuterated 14,15-EET to 14,15-DHET in HEP-G2 cell preparations was measured via LC-MS/MS. HEP-G2 cells express high amounts of sEH which renders them an ideal tool to investigate enzyme activity in a less artificial environment (Figure 5B and Table 3). All CysLT1RAs tested in this study inhibited the formation of 14,15-DHET from the corresponding epoxide with IC_50_ values of 0.82 ± 0.24 μM, 1.95 ± 0.30 μM, and 14.11 ± 3.22 μM for zafirlukast, montelukast, and pranlukast, respectively.

**Table 3:** Inhibition of sEH by CysLT1RA.

	sEH rec.	sEH (HEP-G2)	CysLT_1_R
		
	IC_50_ [μM]		[nM]
Zafirlukast	**1.97** ± 0.08 μM	**0.82** ± 0.24 μM	5
Montelukast	n.d.	**1.95** ± 0.30 μM	5
Pranlukast	**8.86** ± 0.19 μM	**14.11** ± 3.22 μM	4


*sEH activities were determined with recombinant enzyme as well as HEP-G2 cell lysates. For determination of the activity of recombinant sEH, a fluorescence-based assay using PHOME as substrate was employed. In contrast, sEH activity in HEP-G2 cell lysates was measured by conversion of deuterated 14,15-EET into the corresponding diol by LC-MS/MS technique. Results are shown SEM of three independent experiments. n.d., not determined due to autofluorescence of the compound; rec, recombinant enzyme*.

## Discussion

In the present report, we have investigated the sEH inhibitory as well as the PPAR activating potential of the marketed CysLT1RAs montelukast, pranlukast and zafirlukast. As expected, the chemically distinct scaffolds of these compounds resulted in different interaction profiles on the investigated targets. Zafirlukast turned out to be a dual modulator of human sEH and PPARγ. Montelukast and pranlukast were primarily sEH inhibitors.

CysLT_1_ receptor antagonists have been developed for the treatment of atopic diseases such as asthma and allergic rhinitis. Displaying low nanomolar inhibitory activity to their target receptor these well-tolerated compounds were originally designed to inhibit the vasodilatory and bronchoconstrictory activities of cysteinyl leukotrienes. The anti-inflammatory properties of this compound class are well documented and therapy is thought to have few side-effects and therefore is considered safe. Recently, it has been shown that asthmatic patients taking montelukast display lower levels of blood lipids and CVD-associated inflammatory biomarkers (Hwang et al., 2013; Ibrahim et al., 2014; Hoxha et al., 2017). In accordance, a retrospective Swedish nationwide study suggested that regular intake of montelukast reduces the risk for recurrent stroke and myocardial infarction (Allayee et al., 2007; Ingelsson et al., 2012). In line with these observations, higher doses of montelukast and zafirlukast are effective in several animal models of CVDs were they showed anti-atherosclerotic effects, protected from myocardial infarction and renal perfusion injury in rabbits, rats and mice (Sener et al., 2006; Jawien et al., 2008; Mueller et al., 2008; Ge et al., 2009; Liu et al., 2009). In addition, during treatment with zafirlukast (20 mg, twice daily) rare cases of hypoglycemic events were observed and it was shown recently that the compound increases insulin secretion from pancreatic β cells (Calhoun et al., 1998; Hwang et al., 2018). These findings concerning the beneficial effects on cardiovascular and diabetic disorders clearly argue for an additional, CysLT-unrelated, mechanism of action which can be exploited for therapy in the future. Indeed, several publications could already show that montelukast, pranlukast as well as zafirlukast display an interesting polypharmacological profile. Already described off-target effects are inhibition of additional pro-inflammatory targets such as the PGE_2_ down-stream synthase mPGES-1, 5-lipoxygenase, cAMP phosphodiesterases and NFκB (Ramires et al., 2004; Anderson et al., 2009; Fogli et al., 2013; Kahnt et al., 2013; Theron et al., 2014; Hoxha et al., 2017).

The combination therapy of sEH inhibition with PPARγ agonism effectively lowers blood pressure, reduces systemic glucose, triglyceride and free fatty acid levels and is also renoprotective in animal models (Imig et al., 2012; Hye Khan et al., 2018). sEH as well as the PPAR receptors interact with a number of oxidized arachidonic acid metabolites, so-called eicosanoids. Therefore, interaction with eicosanoid mimetic compounds such as CysLT1RA suggests itself. Due to the fact that the already published off-target activities of the marketed CysLT1RAs do not sufficiently explain the beneficial effects on cardiovascular outcome and insulin secretion in man as well as their efficacy in various animal models of atherosclerosis and the MetS, we were interested if these compounds interact with sEH and PPAR isoform activities.

sEH is an enzyme that hydrolyzes cytochrome P450-derived arachidonic acid epoxides (epoxyeicosatrienoic acids, EETs) with potent vasoprotective activities into their corresponding vicinal diols (dihydroxyeicosatrienoic acids – DHETs). Upon this conversion, the beneficial effects of EETs such as vasodilation, inhibition of platelet aggregation, promotion of fibrinolysis and reduction of vascular smooth muscle cell proliferation are lost. Accordingly, it has been demonstrated in numerous studies that inhibition of sEH is antihypertensive, organ protective and has beneficial effects on glucose metabolism (Fleming, 2014). According to the results presented in this study, all CysLT1RAs investigated inhibit human sEH. Montelukast and zafirlukast showed IC_50_ values in the nanomolar to low micromolar range which can be easily achieved with therapeutic dosage in humans as both compounds reach low micromolar plasma levels upon frequent dosing. This might explain the beneficial effects of montelukast on CVD-associated events and also raises this possibility for zafirlukast. The IC_50_ value for sEH inhibition of pranlukast was higher and might not be achieved *in vivo*.

We investigated the influence of the CysLT1RA on activation of the PPAR isoforms, γ, α, and δ. While the α and δ isoforms were not influenced by any of the tested compounds, montelukast and zafirlukast activated the PPARγ reporter construct in our study. Zafirlukast showed potent maximal activation of the receptor compared to the reference agonist pioglitazone (∼150%) with an IC_50_ of 2.49 μM whereas montelukast showed a reduced overall receptor activation compared to the control with an IC_50_ of 1.17 μM. Then, we investigated the influence of both compounds on PPARγ activation further by studying the differentiation of 3T3-L1 cells into adipocytes followed by PPARγ target gene expression analysis. Zafirlukast dose-dependently triggered 3T3-L1 differentiation judging from the lipid accumulation data and the target genes investigated. Surprisingly, the extent of lipid accumulation and target gene upregulation was lower than anticipated. Montelukast did not induce cell lipid accumulation and showed only weak effects on some target genes investigated. Nevertheless, both montelukast and low dose zafirlukast potently upregulated the metabolic activity of 3T3-L1 cells comparable to the rosiglitazone control. In contrast, high concentrations of zafirlukast (5 and 10 μM) had no influence on mitochondrial activity.

PPARγ is a member of the PPAR nuclear receptor family and plays a key role in adipogenesis, lipid metabolism, glucose homeostasis and anti-inflammatory processes (Tontonoz and Spiegelman, 2008). It is therefore targeted for the treatment of type II diabetes. Activation of this nuclear receptor by thiazolidinedione (TZD) compounds such as pioglitazone and rosiglitazone influences insulin action and blood-glucose levels (Staels and Fruchart, 2005). Unfortunately, the clinical use of TZDs is limited due to excessive weight gain, edema formation and an elevated osteoporosis incidence in patients. Although the equilibration of blood glucose levels reduces microvascular complications under TZD treatment, these compounds are only poorly effective on the occurrence of macrovascular events (Rohatgi and McGuire, 2008; Ahmadian et al., 2013). In contrast to endogenous PPARγ ligands which display moderate and selective induction of certain PPARγ target genes, TZDs upregulate target gene expression with high potency in a global fashion. This is thought to be the cause for many of the adverse events seen under TZD treatment. At present, development of new PPARγ activators focuses on compounds that selectively fine tune PPARγ activity instead of exerting full blown agonism. These new modulators differentially influence cofactor recruitment or act as partial agonists that influence expression in a target gene and cell type dependent manner (Wright et al., 2014; Garcia-Vallvé et al., 2015).

In our hands, zafirlukast displayed a high maximal induction of the reporter gene construct in HEK-293T cells but its effect on 3T3-L1 lipid accumulation and PPARγ target gene expression was way lower than anticipated from its reporter gene data. Due to this, we hypothesized that zafirlukast might additionally influence post-translational modifications of PPARγ and thus cofactor recruitment in adipocytes. PPARγ binds a number of cofactors depending on the target gene. For this, the receptor can be modified by phosphorylation of various serine residues which influence receptor activity und target gene transcription by altering cofactor recruitment. Among these modifications is the CDK5-mediated phosphorylation of PPARγ that is triggered under pro-inflammatory conditions. This modification does not suppress the transcriptional activity of the receptor in general along with its overall adipogenic capacity. Instead, it modulates the recruitment of co-regulators leading to a dysregulation of various genes, among them adiponectin, whose expression is altered under chronic inflammatory conditions such as obesity. Interestingly, this phosphorylation can be blocked by anti-diabetic PPARγ ligands, such as rosiglitazone (Choi et al., 2011). Indeed, zafirlukast was able to completely revert this phosphorylation in TNFα treated 3T3-L1 adipocytes which shows that the compound most probably interferes with the binding of cofactors. This might explain the differing extent of target gene upregulation compared to rosiglitazone and the attenuated mitochondrial activity in cells treated with higher concentrations of zafirlukast.

Owing to the phosphorylation data, we were interested in the direct influence of montelukast and zafirlukast on PPARγ coactivator recruitment. For this, we used an assay system in which the recruitment of the fluorescence-tagged PPARγ coactivator CBP to the PPARγ-LBD was assessed by time-resolved FRET technique in presence and absence of rosiglitazone. Zafirlukast did not induce CBP recruitment itself. Instead, the compound potently antagonized the rosiglitazone induced cofactor recruitment in a non-covalent manner with an IC_50_ of 4.7 μM. Montelukast weakly antagonized the rosiglitazone induced CBP recruitment. At higher concentrations the compound led to an elevation of the FRET signal probably due to unspecific protein aggregation. These results clearly argue for an interesting PPARγ modulatory role of zafirlukast rather than full agonism. In addition, this explains both the differing activation potencies of zafirlukast for adiponectin compared to the TZD control and the absence of 3T3-L1 mitochondrial activation in cells treated with high concentrations of the compound. Montelukast treatment was not able to trigger lipid accumulation in 3T3-L1 pre-adipocytes which fits to its weak PPARγ induction measured in the reporter gene assay experiments. Nevertheless, it potently elevated the mitochondrial activity of these cells. If this is due to its weak interference with PPARγ cofactors and/or sEH antagonism or another off-target effect of the compound remains to be investigated in the future.

**FIGURE 6:**
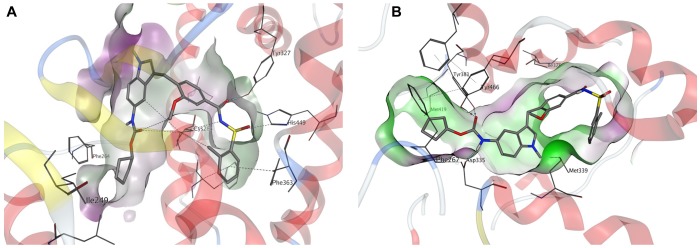
Molecular docking of zafirlukast to human PPARγ and sEH. **(A)** Proposed binding mode of zafirlukast (gray sticks) to the PPARγ ligand binding domain – the acidic acyl sulphonamide moiety exhibits directed indications toward the amino acids responsible for activation of PPARγ, while the substituted indole residue occupies a large hydrophobic sub-pocket. **(B)** Proposed binding mode of zafirlukast (gray sticks) to the sEH C-terminal domain – the carbamate moiety interacts with the catalytic triade Tyr383, Tyr466 and Asp335.

We performed *in silico* docking studies to gain further information about the possible binding modes of zafirlukast to its targets. Zafirlukast is based on an indole scaffold which is a privileged heterocycle among fatty acid mimetics (Proschak et al., 2017). Different functional moieties are probably responsible for the interaction with the targets investigated in this study: Inhibition of sEH is most probably caused by the presence of a carbamate function, which acts as an epoxide mimetic and interacts with the enzyme’s catalytic triad. This carbamate function is enclosed by two lipophilic moieties that fit well into the lipophilic pockets adjacent to the catalytic center of sEH. Indeed, molecular docking revealed that the linear shape of zafirlukast fits well into the binding site of human sEH (Figure 6B). While the carbamate moiety is mainly responsible for sEH binding, the acidic acyl sulfonamide linked to a lipophilic region on the opposite side of the molecule is a characteristic pharmacophore of PPARγ agonists. Indeed, our molecular docking studies suggested that this moiety is able to interact with four amino acid residues responsible for stabilization of the AF-2 helix in PPARγ (Figure 6A). Of note, this region is important for the receptor’s cofactor recruitment and activation.

Taken together, zafirlukast is a marketed CysLT_1_ receptor antagonist with additional anti-inflammatory properties such as inhibition of pro-inflammatory PGE_2_ that also exhibits sEH inhibitory and PPARγ modulatory properties at pharmacologically relevant concentrations. Especially in the inflammatory environment, the simultaneous inhibition of sEH and moderate activation of PPARγ should lead to downregulation of NFκB target genes and TGF-β1/Smad3 as well as resolution of inflammation by the induction of anti-inflammatory and pro-resolving mediators resulting in inhibition of leukocyte influx into the inflamed tissue and promotion of alternatively activated macrophages (Kim et al., 2014; Croasdell et al., 2015). This has to be confirmed in follow-up studies employing animal models of acute and chronic inflammation which can provide new insights into how the crosstalk between sEH and PPARγ is influenced by zafirlukast. In addition, zafirlukast is an excellent starting point for the further development of a compound class displaying superior polypharmacology for the treatment of chronic inflammatory diseases such as the MetS. High plasma protein binding has been observed for zafirlukast and peak serum levels achieved after a single oral dose of 20 mg are about 1 μM (Brocks et al., 1996; Knorr et al., 2001; Karonen et al., 2012). This is pretty close to the EC_50_ and IC_50_ values assessed for PPARγ and sEH in this study but might hamper the achievement of full activity during therapy. Due to zafirlukast’s equipotent target profile and excellent pharmacokinetic properties, derivatives of zafirlukast are bearing the potential to be further optimized through SOSA (selective optimization of side activity) strategy for various indications (Imig et al., 2012; Blöcher et al., 2016a,b). Indeed, we have recently developed a number of zafirlukast derivatives and successfully accomplished this task by developing a compound with increased bioactivity (Schierle et al., 2018). This lead compound showed improved inhibition of human sEH as well as activation of PPARγ while it displayed favorable pharmacokinetic properties *in vivo*.

## Data Availability

All datasets generated for this study are included in the manuscript and/or the Supplementary Files.

## Author Contributions

TG, OD, SW, DM, CA, EB, RK, LW, TS, JH, and AK performed the experiments. AK, TM, GG, MS-Z, DS, and EP contributed to the conception and design of the study as well data interpretation and analysis. AK and EP wrote the manuscript. All authors contributed to manuscript revision, read and approved the submitted version.

## Conflict of Interest Statement

The authors declare that the research was conducted in the absence of any commercial or financial relationships that could be construed as a potential conflict of interest.
