# Supplementary material for: Zafirlukast Is a Dual Modulator of Human Soluble Epoxide Hydrolase and Peroxisome Proliferator-Activated Receptor γ
Source: Front Pharmacol. 2019 Mar 20;10:263. doi: 10.3389/fphar.2019.00263 (PMC6435570; doi:10.3389/fphar.2019.00263)
Supplement: Supplementary file 1 [file Presentation_1.pptx]

## Slide 1
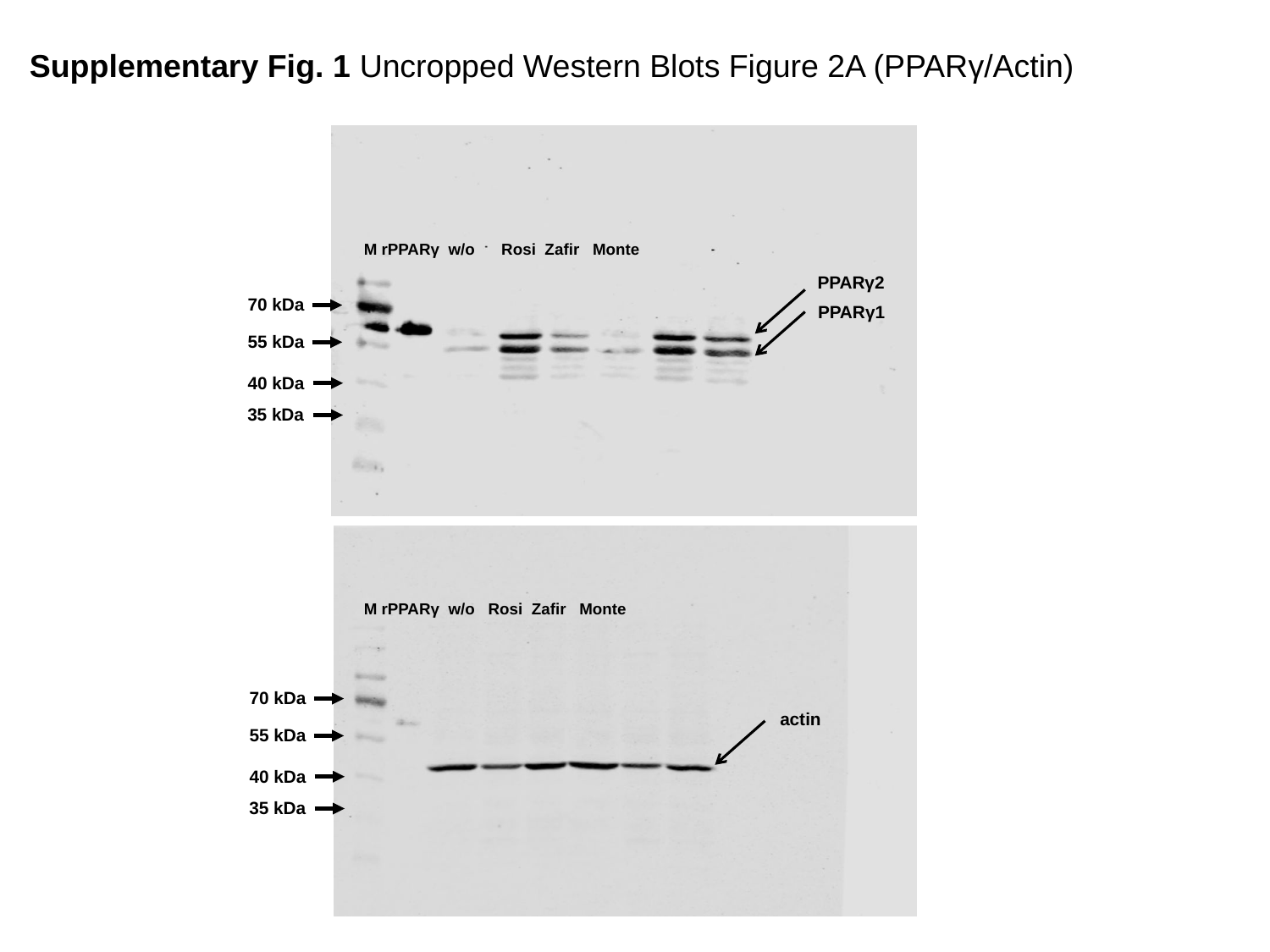

Supplementary Fig. 1 Uncropped Western Blots Figure 2A (PPARγ/Actin)
M rPPARγ w/o Rosi Zafir Monte
PPARγ2
70 kDa
PPARγ1
55 kDa
40 kDa
35 kDa
M rPPARγ w/o Rosi Zafir Monte
70 kDa
actin
55 kDa
40 kDa
35 kDa

## Slide 2
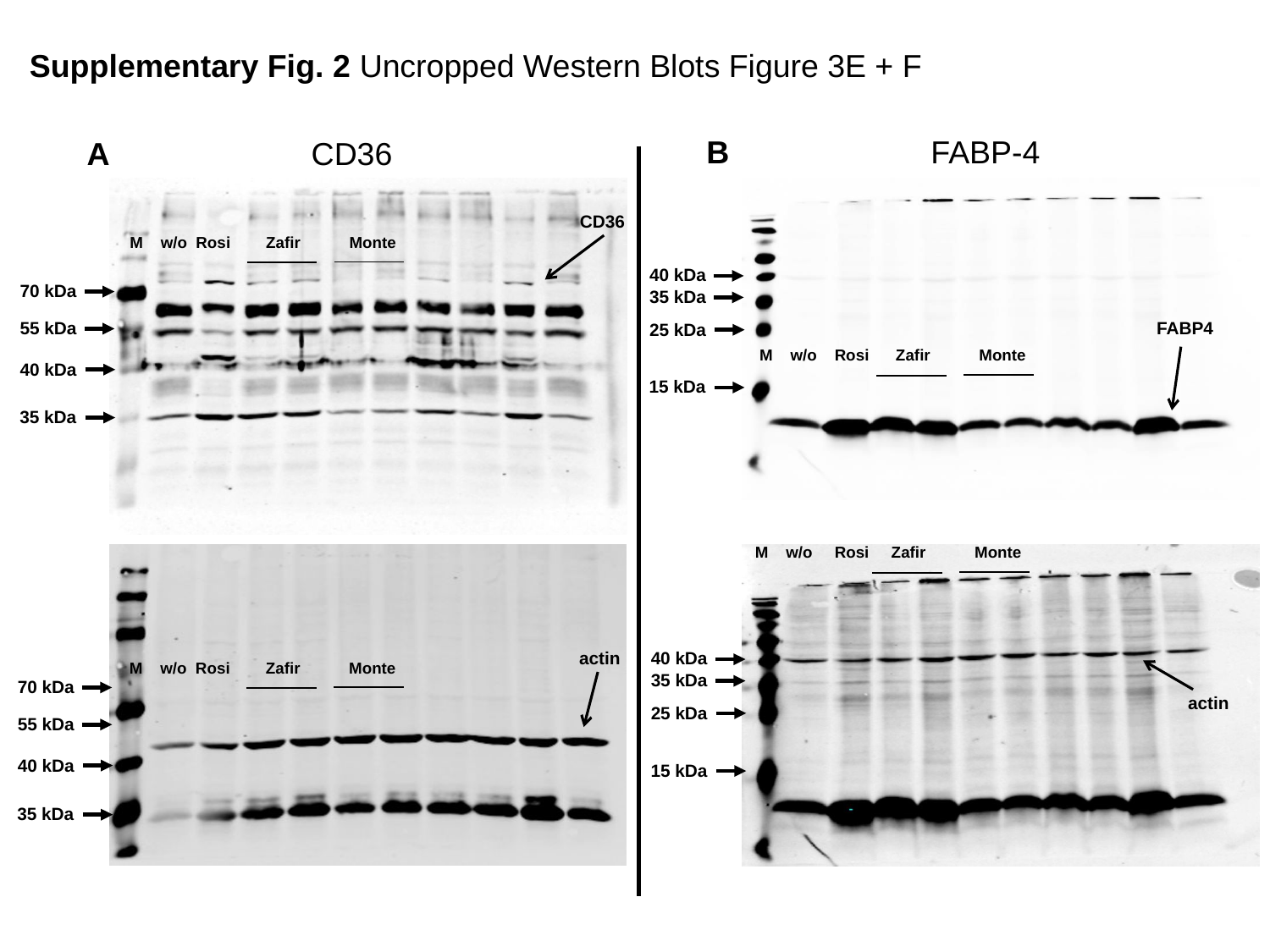

Supplementary Fig. 2 Uncropped Western Blots Figure 3E + F
B	 FABP-4
A	 CD36
CD36
M w/o Rosi Zafir Monte
40 kDa
70 kDa
35 kDa
FABP4
55 kDa
25 kDa
M w/o Rosi Zafir Monte
40 kDa
15 kDa
35 kDa
M w/o Rosi Zafir Monte
actin
40 kDa
M w/o Rosi Zafir Monte
35 kDa
70 kDa
actin
25 kDa
55 kDa
40 kDa
15 kDa
35 kDa

## Slide 3
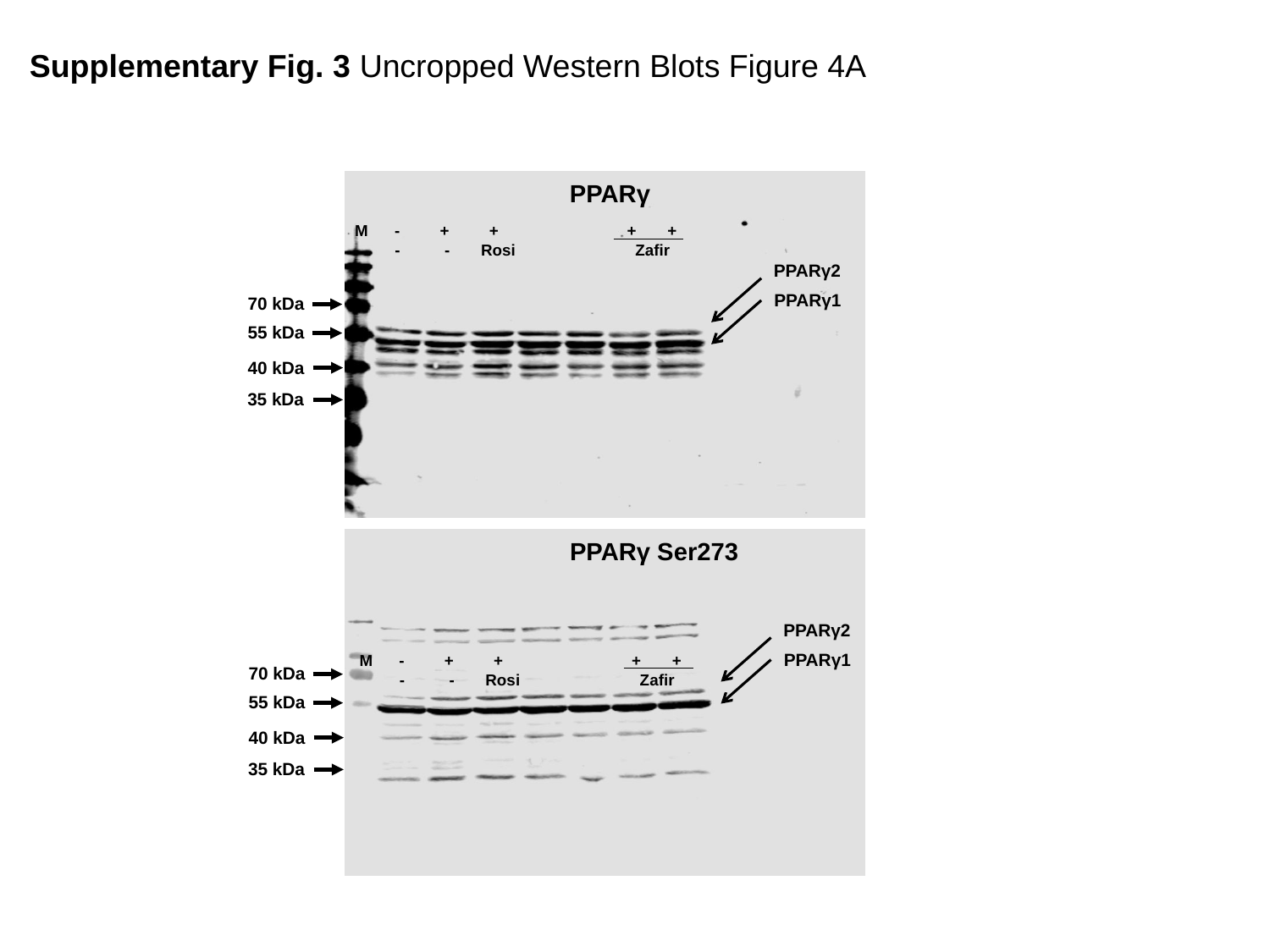

Supplementary Fig. 3 Uncropped Western Blots Figure 4A
PPARγ
M - + + + +
 - - Rosi Zafir
PPARγ2
PPARγ1
70 kDa
55 kDa
40 kDa
35 kDa
PPARγ Ser273
PPARγ2
PPARγ1
M - + + + +
 - - Rosi Zafir
70 kDa
55 kDa
40 kDa
35 kDa
